# Electrostatic Excitation for the Force Amplification of Microcantilever Sensors

**DOI:** 10.3390/s111110129

**Published:** 2011-10-25

**Authors:** Ali Shokuhfar, Payam Heydari, Salman Ebrahimi-Nejad

**Affiliations:** Advanced Materials and Nanotechnology Research Lab, Faculty of Mechanical Engineering, K.N.Toosi University of Technology, Tehran 19991-43344, Iran; E-Mails: Shokuhfar@kntu.ac.ir (A.S.); Ebrahiminejad@dena.kntu.ac.ir (S.E.-N.)

**Keywords:** microcantilever, force amplification, pull-in voltage

## Abstract

This paper describes an electrostatic excited microcantilever sensor operating in static mode that is more sensitive than traditional microcantilevers. The proposed sensor comprises a simple microcantilever with electrostatic excitation ability and an optical or piezoresistive detector. Initially the microcantilever is excited by electrostatic force to near pull-in voltage. The nonlinear behavior of the microcantilever in near pull-in voltage *i.e.*, the inverse-square relation between displacement and electrostatic force provides a novel method for force amplification. In this situation, any external load applied to the sensor will be amplified by electrostatic force leading to more displacement. We prove that the proposed microcantilever sensor can be 2 to 100 orders more sensitive compared with traditional microcantilevers sensors of the same dimensions. The results for surface stress and the free-end point force load are discussed.

## 1. Introduction

Micro- and nano-sensors, especially microcantilever sensors, have attracted considerable interest for recognition of target analytes in biological and chemical and force sensing because of their fast, ease of use and inexpensive properties [1–3]. Despite the promising characteristics of the microcantilever sensor, the low detection limit is a barrier in some applications. For example, in microcantilever based electronic noses, it is difficult to see down to the parts per trillion (10^12^) level, even in highly optimized conditions; whereas the canine nose can work down to the parts per quad (ppq) levels. Consequently, trained dogs currently are the “gold standard” method for analyte detection [4]. As another example, in some cases surface stress microcantilever sensors could not be used to measured low concentrations of bimolecular species [5,6]. These examples show some of the challenges in the development of the applications of microcantilever sensors.

To increase the sensitivity of microcantilever sensors, and therefore, to overcome many of these challenges, a number of methods have been developed [7,8] that can be categorized into: (1) geometric optimization of sensors [9–20]; (2) improvements to the materials used in the fabrication of sensors [21–26]; (3) use of more precise detection methods to detect microcantilever bending [27–29]; (4) improvements to the biological binding in order to increase exerted biological force [30–32]. These categories do not include improvements in readout circuit systems.

Several groups have published reports on the best microcantilever shape in order to achieve maximum sensitivity. Louia and coworkers designed, fabricated, and tested five piezoresistive cantilever configurations to investigate the effect of shape and piezoresistor placement on the sensitivity of microcantilevers [11]. Sukuabol *et al.* [12] used various cantilever shapes and found that the long-base U-shape and inverse-T-shape provide optimum geometries for SU-8 microcantilever sensitivity. Decreasing the thickness of the microcantilevers is another common strategy to increase their sensitivity [13]. By using Finite Element analysis, Chivukula *et al.* [14] have shown that optimizing the device dimensions is useful, to a great extent, in increasing the sensitivity of the device. Another traditional shape optimization method for enhancing the piezoresistive detection sensitivity is based on the stress concentration regions (SCRs) that have been studied by many groups [15–18]. Yang *et al.* [19] designed and fabricated a quad-cantilever sensor with a four-cantilever half-sensitive Wheatstone bridge for improving trace chemical sensing performance. In [20] a double-microcantilever design has been developed to overcome the thermal stress effect. The double microcantilever is composed of a top immobilized microcantilever and a bottom sensing microcantilever. These two microcantilevers could increase the sensitivity by more than two orders of magnitude and minimize the induced thermal effects.

Conventionally, microcantilever sensors are fabricated on a silicon substrate [21]. Recently a polymeric microcantilever is developed which has a much lower Young’s modulus than conventional Si microcantilevers [22,23] and can improve the sensitivity of the sensor. In addition, SiO_2_-based microcantilevers are good candidates having a higher sensitivity because they are made of materials with a lower Young’s modulus (57–70 GPa) than that of Si (170 GPa). For example, Li *et al.* [25,26] showed that piezoresistive microcantilevers made of silicon dioxide are more sensitive than silicon-based microcantilevers. The embedded piezoresistor is made up of single crystal silicon and is fully insulated from the surrounding environment by SiO_2_, resulting in lower electric noise.

The current detection methods in microcantilever biosensors include piezoelectric or piezoresistive detectors for tension sensing and optical or capacitive detectors for displacement measurement. Displacement detectors usually have a higher sensitivity and can respond to very weak input signals. However, the limitation of working in liquid media, which is essential for biological sensors, is the main drawback of displacement detectors. To address this problem, metal-oxide semiconductor field-effect transistors (MOSFET) have been used by Shekhawat and coworkers to achieve a higher sensitivity in microcantilever biosensors [27].

A successful method that has been used for increasing the biological force has been implemented in the force amplified biological sensor under development at the Naval Research Laboratory [32]. This instrument uses forces produced by micron-sized labeled magnetic particles on biological receptor to pull on biomolecules and then the external magnetic field results in piconewton-level forces with sufficient sensitivity to be detected by piezoresistive microcantilevers. Unfortunately, the cost, size, and mechanical complexity of this labeled sensor often preclude their use [32].

Conventional microcantilever sensors work in a linear mode of operation, but recently the nonlinear operation of sensors especially in resonator-based microdevice [33] has received considerable attention. The geometrically nonlinear deformation of beams can be used to improve the signal to noise ratio and robustness for sensors like mass sensor based on parametric resonance [34] and parametric amplification in a microelectromechanical system (MEMS) gyroscope [35].

In this paper a novel microcantilever with electrostatic excitation that is more sensitive than traditional rectangular microcantilevers is proposed. The basic idea comes from the nonlinear electrostatic force:

(1) $$F_{e}=\frac{\in_{0}b\;\;V^{2}}{2{\left(w-g\right)}^{2}}$$

where ∈_0_ = 8.854 × 10^−12^ C.N/m is the permittivity of vacuum, *V* is the applied voltage and *g* is the initial gap between the movable and the ground electrode. In Equation (1) the electrostatic force is inversely related to the distance of the two electrode surfaces. Therefore, if a load on the microcantilever with *b* width reduces the distance between the two electrode surfaces, the electrostatic force increases and hence, the displacement of the microcantilever, *w*, continuously increases. Based on this phenomenon, the electrostatic force can amplify others sources of load and so, very low forces or surface stresses can be observable. The proposed microcantilever sensor that is similar to a microswitch could be fabricated by most micromachining processes. An advantage of this sensor over the microcantilever is that this approach can amplify the input load without the need for labeling. In addition, many other methods for increasing the sensitivity of microcantilever sensors can be simultaneously incorporated into the proposed method.

In the following section, the nonlinear Euler-Bernoulli beam equations for the proposed microcantilever sensor have been obtained. The proposed model has been solved by Green’s function method, and the verification of results for pull-in voltage and displacement under electrostatic force has been performed. In Section 3, the numerical analysis and comparison of the sensitivity of traditional microcantilever sensors and the proposed electrostatic excited microcantilever sensor has been discussed. In addition, the influence of geometrical factors including the initial gap, width, length and thickness on the sensitivity of the microcantilever sensor has been explored. We close the paper with concluding remarks in Section 4.

## 2. Mathematical Theory

**Figure 1.**
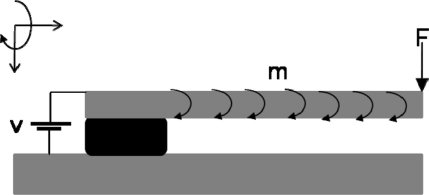
Schematic representation of an electrostatic excited microcantilever sensor.

An electrostatic excited microcantilever sensor is composed of a microcantilever beam separated by a dielectric spacer from a fixed ground plane (Figure 1). Based on the operation principle of the proposed sensor, the microcantilever deflects toward the underlying fixed ground plane due to attractive electrostatic forces. At near “pull-in” voltage, the microcantilever sensor which is subjected to external load (force or moment) can amplify the displacement.

For performance analysis of the proposed sensor, two different applications of microcantilevers are dealt with here. The tip force applied to the microcantilever in Figure 1 has been used for modeling the first application, which is the original function of microcantilevers as a force or deflection sensor, as seen in atomic force microscopes (AFMs). The second application of the proposed sensor is in biosensing, where isotropic surface stresses are encountered. Based on Yin Zhang’s assumption [36], the surface stress effect is modeled as a distributed moment *m* applied along the microcantilever (see Figure 1). The following relation between the surface stress, *σ* and the uniformly distributed bending moment *m* along the microcantilever can be established as:

(2) $$m=\frac{\sigma \;b\;t}{2\;\times \;L}$$

To study the nonlinear behavior of the electrostatic excited microcantilever sensor, a beam model is derived for the microcantilever of length *L* with a uniform cross section of width *b* and thickness *t*. Based on Euler-Bernoulli’s beam theory the governing Equation may be written as:

(3) $$\mathrm{EI}\frac{d^{4}w}{{\mathrm{dx}}^{4}}=F_{e}+\left(\frac{m}{E\;I}+\frac{F}{E\;I}\right)\delta (x-L)$$

and the associated boundary conditions are:

(4a) $$w(0)=\frac{\mathrm{dw}}{\mathrm{dx}}(0)=0$$

(4b) $$\frac{d^{2}w}{{\mathrm{dx}}^{2}}(L)=0$$

(4c) $$\frac{d^{3}w}{{\mathrm{dx}}^{3}}(L)=0$$

where *F_e_* is the electrostatic force per unit length of the microcantilever, formulated in Equation (1) *w* is the deflection of the microcantilever, *x* is the position along the microcantilever measured from the clamped end, *E* is the Young’s modulus, and *I* is the microcantilever second moment of area, which, for a rectangular cross section, is:

(5) $$I=\frac{b\times t^{3}}{12}$$

For convenience, the model is formulated in a nondimensional form, by introducing the nondimensional variables:

(6) $$u=\frac{w}{g},z=x/L$$

The following nondimensional equation is obtained:

(7) $$\frac{d^{4}u}{{\mathrm{dz}}^{4}}=F(z)=\frac{\in_{0}b\;\;V^{2}L^{4}}{2\;E\;I\;\;g^{3}{\left(1-u(z)\right)}^{2}}+\left(\frac{{\mathrm{FL}}^{3}}{g\;E\;I}+\frac{{\mathrm{mL}}^{3}}{g\;E\;I}\right)\delta (z-1)$$

and the associated boundary conditions are:

(8a) $$u(0)=\frac{\mathrm{du}}{\mathrm{dz}}(0)=0$$

(8b) $$\frac{d^{2}u}{{\mathrm{dz}}^{2}}(1)=0$$

(8c) $$\frac{d^{3}u}{{\mathrm{dz}}^{3}}(1)=0$$

According to the definition of the nondimensional variables, physically meaningful solutions exist in the region *0 < u < 1*, where *u* is the deflection of the cantilever tip. Integral equation representations are useful for understanding the response of a system to a concentrated load, since from the theoretical point of view, the solution for an arbitrary load can be constructed using only the known load and the solution for a concentrated load [37]. The concentrated load at *z* = ξ is modeled using the Dirac delta function δ(z – ξ). Replacing *F*(*z*) with δ(z – ξ) and *u* with *G* in Equation (7), one obtains:

(9) $$\frac{d^{4}G}{{\mathrm{dz}}^{4}}=\delta (z-\xi )$$

which models a microcantilever beam with a concentrated load at *z* = ξ. The solution to this problem, called the Green’s function is:

(10) $$G=\{\begin{array}{ll} a_{0}z^{3}+a_{1}z^{2}+a_{2}\;z+a_{3} & 0\leq z<\xi \\ a_{0}z^{3}+a_{1}z^{2}+a_{2}\;z+a_{3} & \xi <z\leq 1 \end{array}$$

The coefficients *a_i_* and *b_i_* (*i = 0,1,2,3*) in Equation (10) are unknown constants. The boundary conditions (fixed at *z = 0* and free at *z =1*) are imposed:

(11a) $$G(0)=\frac{\mathrm{dG}}{\mathrm{dz}}(0)=0$$

(11b) $$\frac{d^{2}G}{{\mathrm{dz}}^{2}}(1)=0$$

(11c) $$\frac{d^{3}G}{{\mathrm{dz}}^{3}}(1)=0$$

Equation (10) still has four unknown constants to be determined from the continuity of the solution and its first and second derivatives at *n*, *i.e.*,

(12a) $$G\left(\xi^{-}\right)=G\left(\xi^{+}\right)$$

(12b) $$\frac{\mathrm{dG}}{\mathrm{dz}}\left(\xi^{-}\right)=\frac{\mathrm{dG}}{\mathrm{dz}}\left(\xi^{+}\right)$$

(12c) $$\frac{d^{2}G}{{\mathrm{dz}}^{2}}\left(\xi^{-}\right)=\frac{d^{2}G}{{\mathrm{dz}}^{2}}\left(\xi^{+}\right)$$

(12d) $$\frac{d^{3}G}{{\mathrm{dz}}^{3}}\left(\xi^{+}\right)-\frac{d^{3}G}{{\mathrm{dz}}^{3}}\left(\xi^{-}\right)=1$$

As the deflection of a microcantilever beam with concentrated load of unit strength at point ξ is:

(13) $$G=\{\begin{array}{ll} \left(-\frac{1}{6}\right)z^{3}+\left(\frac{\xi }{2}\right)z^{2} & 0\leq z<\xi \\ \left(\frac{\xi^{2}}{2}\right)z-\frac{\xi^{3}}{6} & \xi <z\leq 1 \end{array}$$

Now, the derived Green’s function is used to construct the solution to our nonuniformly distributed loading problem. Multiplying Equation (9) by *u*, Equation (7) by *G*, subtracting the two Equations, and integrating from *z* = *0 to z* = *1*, one may obtain:

(14) $$\int_{0}^{1}\left(G\frac{d^{4}u}{{\mathrm{dz}}^{4}}-u\frac{d^{4}G}{{\mathrm{dz}}^{4}}\right)\mathrm{dz}=\int_{0}^{1}(\mathrm{FG}-u\delta )\mathrm{dz}$$

This is the integral representation of the nonlinear differential Equation (7). In this way, the Green’s function is used to turn the nonlinear differential Equation (7) into the nonlinear integral Equation (14). Integrating the left side of Equation (14) by parts and applying the boundary conditions Equations (8) and (11), all contributions from these terms vanish and one is left with noting that *G(z, ξ)* is a symmetric function of *z* and ξ, one may rename the variables and write:

(15) $$u(z)=\int_{0}^{1}F(z,\xi ).G(z,\xi )d\xi$$

The closed-form solution of the deflection of the microcantilever tip (*i.e.*, the maximum deflection) is:

(16) $$u(z=1)=\int_{0}^{1}F(z=1,\xi ).G(z=1,\xi )\;d\xi$$

which is obtained by substituting *z = 1* in Equation (15). No solution is possible without assuming a shape function for *u*(*ξ*). The deflection of the microcantilever can be approximated by the following quadratic function [38] satisfying the geometrical boundary conditions:

(17) $$u(\xi )=u_{0}\xi^{2}$$

Substituting Equation (17) into Equation (16) leads to:

(18) $$u_{0}=\int_{0}^{1}\left[\frac{\in_{0}\;b\;\;V^{2}L^{4}}{2\;E\;I\;g^{3}{\left(1-u(\xi )\right)}^{2}}+\left(\frac{{\mathrm{FL}}^{3}}{g\;E\;I}+\frac{{\mathrm{mL}}^{3}}{g\;E\;I}\right)\delta (\xi -1)\right].\left[\left(\frac{1^{2}}{2}\right)\xi -\frac{1^{3}}{6}\right]d\xi$$

Evaluating the integrals on the right side of Equation (18), and inserting *I* from Equation (5) into Equation (18) one obtains:

(19) $$\begin{array}{l} u_{0}=\frac{\in_{0}V^{2}L^{4}}{E\;h^{3}g^{3}u_{0}^{2}}\left[\frac{\log\left(\frac{1}{1-u_{0}}\right)}{2}-\frac{1}{2\left(1-u_{0}\right)}+\frac{1}{2}+\frac{3}{2\left(\frac{1}{u_{0}}-1\right)}+\frac{3\sqrt{u_{0}}\log\left(\frac{1-\sqrt{u_{0}}}{1+\sqrt{u_{0}}}\right)}{4}\right]\\ \;\;\;\;\;\;\;\;\;\;\;\;\;\;\;\;\;\;\;\;\;\;\;\;\;\;\;\;\;\;\;\;+\frac{4FL^{3}}{\mathrm{gEb}h^{3}}+\frac{4mL^{3}}{\mathrm{gEb}h^{3}} \end{array}$$

By solving Equation (19) via Newton’s method or any other method for solving nonlinear algebraic equations, the nondimensional microcantilever tip deflection *u_0_* is obtained, which is due to electrostatic pre-excitation force, tip applied force and distributed moment. The second and third terms on the right hand side of Equation (19) are the well known solutions of microcantilever deformation equation without electrostatic excitation. We can separate this part of the solution as:

(20) $$u_{\mathrm{st}}=(F+M)\frac{4\;L^{3}}{\mathrm{gEb}h^{3}}$$

Because the applied tip force and distributed moment have similar influences on microcantilever displacement, as seen in Equation (20), the rest of the paper only investigates the effect of the applied tip force.

To ascertain the validity of the proposed model, Table 1 compares the experimental, analytical and simulation results for the deflection of a microcantilever below the pull-in voltage under electrostatic pre-exciting force. Table 2 clearly shows that the deflection results of the present work agree with the experimental results better than the analytical results of [39] for the same system configuration. In addition, the pull-in voltage obtained experimentally in [39] is 68.5 V, which is close to the estimated pull-in voltage (69.6 V) using the proposed model. Clearly the pull-in results of the present work are in better agreement with the experimental results in comparison to the analytical results of [40] and [41] which are 66.4 V and 66.78 V, respectively. A comparison among the results shows that the proposed modeling and simulation results have good accuracy compared with other references. Now, we can use this model for determining the performance of the proposed electrostatic excited microcantilever sensor.

Based on the concept development in this paper, the external load applied on the microcantilever sensor in the presence of nonlinear electrostatic excitation should be amplified. To confirm the proposed idea, the amplification factor, *AF*, is defined as:

(21) $$\mathrm{AF}=\frac{u_{0}-u_{\mathrm{es}}}{u_{\mathrm{st}}}$$

The amplification factor demonstrates the ratio of the proposed electrostatic pre-excited microcantilever deflection to simple microcantilever sensor deflection due to tip force or distributed moment. In Equation (21) *u_es_* is the pre-excited nondimensional tip deflection due only to electrostatic excitation. For the calculation of *u_es_*, the external applied tip force and distributed moment should be set to zero, and then Equation (19) be solved for *u_0_* by Newton’s method or any other method used for solving the nonlinear algebraic equation. Therefore, the numerator of Equation (19) is the total nondimentional microcantilever deformation, *u_0_* (due to electrostatic pre-exciting, the tip force and distributed moment) minus the nondimentional microcantilever deformation, *u_es_* (only due to electrostatic pre-exciting). This term describes the after pre-exciting deflection of microcantilever due to tip force or distributed moment. The denominator of Equation (19) is the nondimentional deflection of simple microcantilever without electrostatic pre-exciting calculated using Equation (20).

## 3. Results and Discussion

### 3.1. Influence of Electrostatic Excitation on the Force Amplification

Table 2 lists the mechanical and geometric properties of the microcantilever used in the rest of paper as a reference microcantilever. The mechanical properties of SU-8 have been used for modeling. The amplification factor for the five different tip forces *vs.* the various applied voltages can be seen in Table 3. An increase in the applied voltage raises the amplification factor. Furthermore, at low applied voltage levels, the amplification factor for various forces is linear, but nonlinearity starts showing its effects as the applied voltage increases to near pull-in voltage. The nonlinearity of the sensor response can be accommodated by nonlinear calibration methods which have been greatly advanced these days [42]. On the other hand, in many cases only the detection of the presence of a particular material may be adequate. For instance, in an application such as finding illegal drugs for which trained dogs are utilized for quantitative detection, the important subject is the minimum detection quantity. In these conditions, where the presence of a particular material is important but not its precise amount, the proposed electrostatic excited microcantilever sensor can be suggested as a rival for trained dogs.

**Figure 2.**
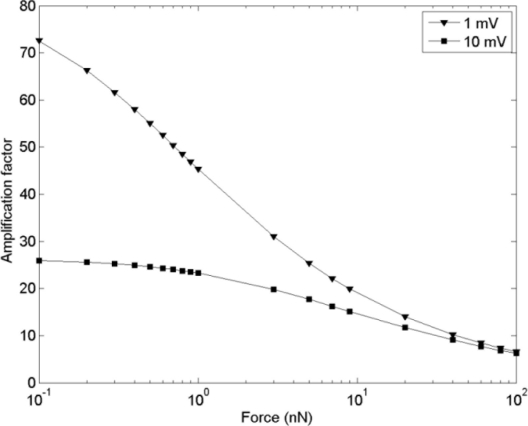
Amplification factor *vs.* various applied force. The applied voltage is 1 mV and 10 mV under the pull-in voltage.

In order to increase the amplification factor, the applied voltage should be closer to the pull-in voltage. Figure 2 shows the amplification factor variation for the reference microcantilever sensor *versus* the applied force. Since the pull-in measurement can be done with μV accuracy [43], we apply voltage to within 1 mV and 10 mV of the pull in voltage. For these two applied voltages the amplification factor is higher for a smaller applied force. As an example for the reference microcantilever, the amplification factor can intensify the 0.1 nN force by a factor of 74.

### 3.2. Size-Dependent Amplification Factor in Electrostatic Exited Microcantilever Sensor

**Figure 3.**
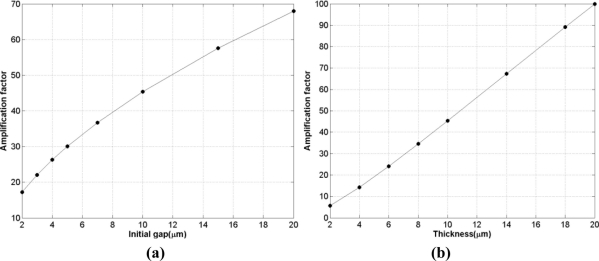

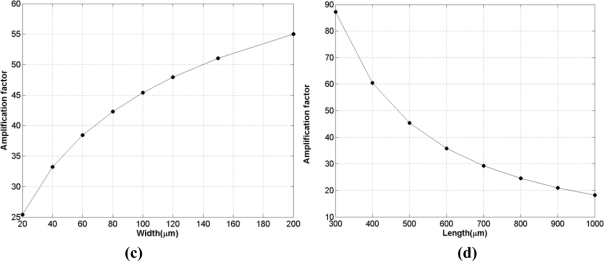
The relationship between amplification factor and the geometric parameters of proposed microcantilever sensor. The applied force is equal to 1 nN and the reference microcantilever data has been used for simulation. The excitation voltage is 1 mV below the pull-in voltage.

This section has been devoted to studying the effect on the amplification factor of the proposed electrostatic excited microcantilever of the variation of four geometric parameter variations which include width, thickness, length and initial gap. First of all, for investigating the influence of initial gap on the amplification factor, numerical simulation has been done based on the data obtained from the reference microcantilever with an the initial gap that changes from 2 μm to 20 μm. The value of excitation voltage in simulation changes corresponding to the initial gap. Table 4 shows the pull-in voltage *versus* the different initial gaps. The excitation voltages are 1 mV under the corresponding pull-in voltage. For instance, at the 10 μm initial gap the pull-in voltage of the reference microcantilever is equal to 49.2159 V; hence the applied voltage should be 49.2149 V. The simulation results for 1 nN applied tip force in Figure 3(a) illustrate how an increase of initial gap leads to a rise in the amplification factor. The reason for this increase in the amplification factor is that the nonlinear electrostatic excitation force increases with respect to the tip force. In Equation (19) the deflection due to electrostatic excitation (first term on the right hand side of the equation) is related to the *V^2^/g^3^* factor which increases based on the values of Table 4. In contrast, the applied force decreases with respect to *g* and *V* variations (second term on the left hand side of the equation). Hence, the nonlinear effect and thus the amplification factor are increased.

In order to study the effect of microcantilever sensor thickness on amplification factor, simulations have been performed on the reference microcantilever with the thickness varying from 2 μm to 20 μm based on data of Table 4. From the table, it can be seen that for smaller thicknesses, the static deflection subjected to constant force increases and the pull in voltage decreases. Moreover, in this case, as shown in Figure 3(b), the decrease in pull-in voltage generated by a smaller thickness increases the amplification factor. It can be concluded that when the pull in voltage decreases, the contribution of the nonlinear electrostatic force decreases compared to linear sources of deflection (the applied force or moment); so eventually the amplification factor decreases.

Figure 3(c) depicts the variation in the length of microcantilever *vs.* the amplification factor. As seen, increasing the microcantilever length leads to a reduction in amplification factor, even though it also leads to a larger tip displacement (Table 4). This means that if a shorter microcantilever sensor is needed for any reason involving lack of space or economical reason then the proposed method is much more effective.

As Table 4 shows, unlike the other three parameters width does not affect the pull in voltage. Generally, the deflection due to electrostatic force is independent from microcantilever width; then the small change in pull-in voltage is due to microcantilever deflection generated by applied force. However, the amplification factor is related to ratio of deflection produced by the electrostatic force to that by the applied force. With increases in width, the deflection generated by electrostatic force remains constant whereas deflection due to applied tip force increases; hence the nonlinearity and the amplification factor increases. This means that a reduction in width leads to a decrease in the amplification factor, as shown in Figure 3(d).

## 4. Conclusions

We have presented a novel sensitive microcantilever force sensor with electrostatic excitation in a static mode operation. In order to study the performance of the proposed sensor, the governing equation of the microcantilever sensor subjected to the electrostatic forces is derived as a two-point boundary value problem (BVP). The equation is nonlinear due to the inherent nonlinearity of the electrostatic excitation. The nonlinear differential equation is transformed into a nonlinear integral equation using the Green’s function of the microcantilever. Assuming an appropriate shape function for the microcantilever deflection to evaluate the integrals, closed-form solutions are obtained. Then, the displacement of microcantilever tip and pull-in parameters were computed and compared with experimental and numerical methods. The results prove the validity of the modeling approach for the proposed microcantilever sensor. Using the developed theoretical model, we showed that the proposed microcantilever sensor compared with a traditional microcantilever sensor of the same dimensions can be 2 to 100 times more sensitive in the cases of force sensor or surface stress sensor.

Finally, the effects of width, length, thickness, and the initial gap of the microcantilever sensor on the sensor amplification factor have been studied. Increasing the initial gap, the thicknesses and the width increases the amplification factor. On the other hand, smaller microcantilever lengths generate bigger amplification factors.

## Figures and Tables

**Table 1.** Comparison between analytical and experimental and the present work for microcantilevers deformation under electrostatic force.

**Excitation Voltage [V]**	**Free End Gap (μm)**			**Absolute Error (%)**
	**Analytical [39]**	**Experiment [39]**	**Present work**	
20	90.2	90.5	90.2	0.3
40	84.3	84.6	84.3	0.3
60	71.5	70	70.8	0.8
65	67.5	64	64.3	0.3
67	65	59	60.4	1.5

**Table 2.** Values of the parameters of the reference microcantilever sensor.

**Parameters**	**Magnitude**
*E* (elastic modulus of SU-8)	3.4 GPa
*L* (length of the microcantilever)	500 μm
*b*(width of the microcantilever)	100 μm
*t* (thickness of the microcantilever)	10 μm
*g* (initial gap)	10 μm
*ɛ* (permittivity of air)	8.85 pF/m

**Table 3.** Amplification factor for various applied voltage.

**Applied Force**	**Magnitude**						
	**20 *V***	**40 *V***	**44 *V***	**46 *V***	**47 *V***	**48 *V***	**49 *V***
0.1 nN	1.0498	1.3759	1.645	1.947	2.2368	2.8352	5.9678
1 nN	1.0498	1.3758	1.6447	1.9465	2.2358	2.8327	5.9355
10 nN	1.0497	1.3749	1.6422	1.9411	2.2262	2.8087	5.6496
60 nN	1.0494	1.3698	1.6289	1.9126	2.1764	2.6915	4.6951
100 nN	1.0491	1.3658	1.6187	1.8916	2.1407	2.6132	4.264

**Table 4.** Influence of geometric parameters on pull-in voltage and deformation of proposed microcantilever sensor with electrostatic excitation (*u_0_* × *g*) and without electrostatic excitation (*u_st_* × *g*). The applied force is equal to 1 nN and the reference microcantilever data has been used for simulation.

Initial gap (μm)	2	3	4	5	7	10	15	20
*u_st_ × g* (nm)	1.1364	1.1364	1.1364	1.1364	1.1364	1.1364	1.1364	1.1364
*u_0_ × g* (μm)	1.076	1.6061	2.1357	2.665	3.723	5.3093	7.9519	10.5938
Pull-in Voltage (V)	4.3999	8.0847	12.4485	17.3983	28.8224	49.2159	90.4189	139.2118

Thickness (μm)	2	4	6	8	10	14	16	20
*u_st_ × g* (nm)	142.045	17.756	5.2609	2.2195	1.1364	0.4141	0.1948	0.1420
*u_0_ × g* (μm)	5.3309	5.3318	5.3217	5.3144	5.3093	5.3027	5.2986	5.2971
Pull-in Voltage (V)	4.3358	12.4288	22.8635	35.2120	49.2159	81.5324	118.8658	139.2181

Length (μm)	300	400	500	600	700	800	900	1000
*u_st_ × g* (nm)	0.2455	0.5818	1.1364	1.9636	3.1182	4.6546	6.6273	9.0909
*u_0_ × g* (μm)	5.2972	5.3033	5.3093	5.3151	5.3207	5.3262	5.3314	5.3364
Pull-in Voltage (V)	136.7236	76.9043	49.2159	34.1747	25.1049	19.2178	15.1812	12.2936

Width (μm)	20	40	60	80	100	120	150	200
*u_st_ × g* (nm)	5.6818	2.8409	1.8939	1.4205	1.1364	0.947	0.7576	0.5682
*u_0_ × g* (μm)	5.3069	5.3084	5.3089	5.3091	5.3093	5.3094	5.3095	5.3096
Pull-in Voltage (V)	49.1921	49.207	49.2119	49.2144	49.2159	49.2168	49.2178	49.2188
